# Dual-Step Selective Homoepitaxy of Ge with Low Defect Density and Modulated Strain Based on Optimized Ge/Si Virtual Substrate

**DOI:** 10.3390/ma15103594

**Published:** 2022-05-18

**Authors:** Buqing Xu, Yong Du, Guilei Wang, Wenjuan Xiong, Zhenzhen Kong, Xuewei Zhao, Yuanhao Miao, Yijie Wang, Hongxiao Lin, Jiale Su, Ben Li, Yuanyuan Wu, Henry H. Radamson

**Affiliations:** 1 Institute of Microelectronics, Chinese Academy of Sciences, Beijing 100029, China; xubuqing@ime.ac.cn (B.X.); duyong@ime.ac.cn (Y.D.); xiongwenjuan@ime.ac.cn (W.X.); kongzhenzhen@ime.ac.cn (Z.K.); miaoyuanhao@ime.ac.cn (Y.M.); wangyijie@ime.ac.cn (Y.W.); sujiale@ime.ac.cn (J.S.); 2 School of Integrated Circuits, University of Chinese Academy of Sciences, Beijing 100029, China; 3 Beijing Superstring Academy of Memory Technology, Beijing 100176, China; guilei.wang@bjsamt.org.cn; 4 Research and Development Center of Optoelectronic Hybrid IC, Guangdong Greater Bay Area Institute of Integrated Circuit and System, Guangzhou 510535, China; linhongxiao@ime.ac.cn (H.L.); liben@giics.com.cn (B.L.); 5 School of Information, North China University of Technology, Beijing 100144, China

**Keywords:** CMOS, Ge epitaxy, selective epitaxial growth, compressive strain

## Abstract

In this manuscript, a novel dual-step selective epitaxy growth (SEG) of Ge was proposed to significantly decrease the defect density and to create fully strained relaxed Ge on a Si substrate. With the single-step SEG of Ge, the threading defect density (TDD) was successfully decreased from 2.9 × 10^7^ cm^−2^ in a globally grown Ge layer to 3.2 × 10^5^ cm^−2^ for a single-step SEG and to 2.84 × 10^5^ cm^−2^ for the dual-step SEG of the Ge layer. This means that by introducing a single SEG step, the defect density could be reduced by two orders of magnitude, but this reduction could be further decreased by only 11.3% by introducing the second SEG step. The final root mean square (RMS) of the surface roughness was 0.64 nm. The strain has also been modulated along the cross-section of the sample. Tensile strain appears in the first global Ge layer, compressive strain in the single-step Ge layer and fully strain relaxation in the dual-step Ge layer. The material characterization was locally performed at different points by high resolution transmission electron microscopy, while it was globally performed by high resolution X-ray diffraction and photoluminescence.

## 1. Introduction

The semiconductor industry started originally with a Ge-based transistor in 1947 [1]. Later, the choice of Ge material turned over to Si due to passivation issues and the feasibility of different processes. It was not until the 1990s when Ge became the focus of interest again in the semiconductor industry due to its electronic and photonic applications [2]. Ge has various advantages. The most promising feature is that Ge has excellent mobility. The electron and hole mobility are two times and four times larger than that of Si, respectively, which can be used to boost the device’s performance by replacing the Si channel [3,4,5,6,7]. The fabrication process of Ge is compatible with the conventional Si-based technology. In addition, the Ge lattice is well-matched with GaAs, which could be used as a buffer layer for the III-V integration in silicon photonics [8]. For other photonic material integration, the Ge buffer layer with high epitaxial quality is also necessary for the growth of GeSnSi materials with direct bandgap properties [9,10,11,12,13]. Growing high-quality Ge on Si is considered an effective approach to make full use of the traditional Si technology and reduce the costs of chips.

A new approach of using a multilayer structure of GeSi/Ge has been proposed for vertical transistors with a gate-all-around (GAA) design. The GeSi layers are etched selectively to Ge in order to create a sub 10 nm channel layer [14,15,16,17].

Selective epitaxy growth (SEG) has been widely used to deposit Ge or GeSi layers in a large variety of devices. As examples, the SEG of GeSi in source/drain areas in MOSFETs or the SEG of Ge as an intrinsic layer in PIN photodetectors have been demonstrated [18]. One problem with the SEG integration is the pattern dependency which leads to a non-uniform Ge (or GeSi) profile over the Si wafers. The main reason behind this problem is the non-uniform consumption of precursor molecules over the dies due to the variation of the exposed Si areas [19,20,21,22]. There are different models to design the chip layout in order to control the pattern dependency. These designs compensate for the exposed Si variations by introducing dummy openings [23,24,25,26,27].

There are two main challenges for the growth of a high-quality Ge epilayer on Si: high lattice mismatch and large thermal expansion coefficients [28]. A tetragonal distortion occurs at the initial state of the growth when the Ge thickness is below the critical thickness (CD). As the thickness exceeds the CD, the strained Ge lattice begins to relax. The dislocation defects generate at the interface and move into the Ge layer. These defects may cause a serious degradation in electronic and optical performance [29,30]. Intensive efforts have been engaged to reduce the defects density in the Ge epilayer. In 1975, Kasper et al. [31] successfully demonstrated the layer-by-layer growth of a Ge/Si_1−x_Ge_x_ (0 < x < 0.15) superlattice and confirmed that three-dimensional island growth would occur if the lattice mismatch was over 0.8%. A graded Ge buffer layer structure was also proposed to reduce the mismatch defects [22,32,33,34,35]. However, this method would result in a large film thickness which is not suitable for the coupling of waveguides in passive devices. A two-step growth method at low and high temperatures was introduced to obtain a Ge epilayer with a low threading dislocation density (TDD) and high surface quality [36]. Long-time cyclic high-temperature annealing was used to decrease the glide dislocations density [37,38,39]. Other methods such as the As-doped LT-Ge buffer [40] and selective area growth [21,41,42] were also investigated to improve the Ge crystal quality. However, the post-annealing process hinders the introduction of the compressive strain into the Ge layer.

This study presents a novel dual-step SEG of Ge to reduce the TDD in Ge layers grown on Si wafers. The defects are depleted in the trench openings and finally diminished in the formed voids on the top. The TDD in Ge is reduced into a 10^5^ cm^−2^ level and the layer is almost fully strain relaxed in contrary to the case when, for example, the Ge layers are directly grown on Si monitor wafers where the TDD is in a 10^7^ cm^−2^ level and Ge is tensile-strained due to the post-annealing treatment (to reduce defects). The mechanism behind this is that threading dislocations are depleted to the oxide walls because of the introduced semicylindrical voids.

## 2. Experimental Details

**Figure 1:**
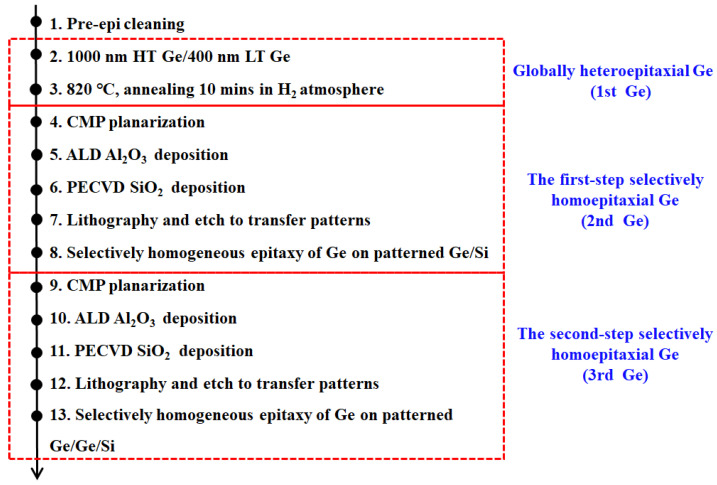
Process flow of the dual-step selective growth of homoepitaxial Ge.

In this study, all the experiments were performed on the 8-inch p-type Si (100) wafers with a resistivity of 0.5–100 Ohm cm. Figure 1 illustrates the completed process flow of the dual-step SEG Ge method. The 1st Ge buffer was grown with a reduced pressure chemical vapor deposition (RPCVD) reactor (ASM Epsilon 2000, Almere, The Netherlands). In the 2nd Ge layer, Al_2_O_3_ was grown with atomic layer deposition (ALD) reactor (TFS200, Beneq, The Netherlands). SiO_2_ trenches were deposited using plasma-enhanced CVD (PECVD) reactor (D250L, Corial, France). It should be mentioned here that the thin Al_2_O_3_ layer was used to stabilize the RPCVD prepared SiO_2_ layer, whose density is lower compared to the thermal furnace method. The detailed growth procedures, from step 1 to step 8, as shown in Figure 1, are described in our previous work which is aimed at preparing a type of Ge/Si film structure with compressive strain that is preferable for p-MOS fabrication [43,44]. After the formation of the 2nd Ge epilayer, we repeated the 4–8 steps to grow the 3rd Ge layer. It should be noted that the patterned SiO_2_ trenches in the 3rd Ge layer were misaligned with the SiO_2_ trenches in the 2nd Ge layer, which will be shown below. This design should be beneficial for blocking the defect in principle.

The cross-section morphology was analyzed by scanning electron microscopy (SEM), HITACHI 5500 Japan. Surface roughness was measured by atomic force microscopy (AFM), Bruker Dimension Icon Inc., Berlin, Germany. High-resolution transmission electron microscopy (HRTEM) by Thermo Fisher Talos, Brno, Czech Republic was employed to determine the crystalline quality [45] and the strain distribution. TEM samples were chosen from target areas in the Ge layers by focused Ga ion beam (FIB micro sampling method) and then polished in an ion milling system using Ar ion. Energy-dispersive spectroscopy (EDS) was employed to determine the element materials of the SEG Ge layers. High-resolution X-ray diffraction (HRXRD) and high-resolution reciprocal lattice maps (HRRLMs) were used to measure the strain changes. The photoluminescence (PL) spectrum was recorded using a 785 nm CW pumping laser, a liquid nitrogen cooled InGaAs detector.

## 3. Results and Discussion

### 3.1. Film Structure and Morphology

**Figure 2:**
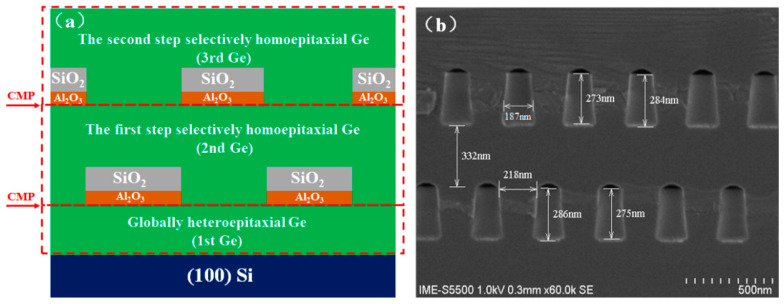
The film structure of dual-step SEG Ge: (**a**) schematic (not to scale), (**b**) cross-section SEM image.

Figure 2a schematically shows the designed dual-step SEG Ge film structure. The structure can be divided into three parts: (1) the globally heteroepitaxial Ge directly grown on an eight-inch Si substrate (first Ge); (2) the first-step selectively homoepitaxial Ge grown on the first Ge (second Ge); and (3) the second selectively homoepitaxial Ge (third Ge). Figure 2b illustrates the cross-section SEM image of the dual-step SEG Ge layer. The boundaries of each unit in the structure are clearly distinguishable. In addition, the sizes of the oxide (SiO_2_ and Al_2_O_3_) trenches have tiny variations. These indicate a high feasibility of the process. The misalignment of the two patterned SiO_2_ trenches was designed to enhance the block of the defects. Regular semielliptical voids are formed at the top of the SiO_2_ trenches as a result of the coalescence of the Ge overgrowth [30]. These voids indicate perfect coalesced Ge layer forms [46,47]. The formation of voids is the critical factor to deplete threading dislocation defects from the SEG Ge layers. In this design, a CMP step is necessary after the global epitaxy to decrease the surface roughness; meanwhile, the second CMP step is after the first SEG to create a uniform Ge thickness over the wafer. This is a necessary step because there is a pattern dependency of the SEG which makes the final Ge thickness different, ranging from the center to the edge of the wafer.

**Figure 3:**
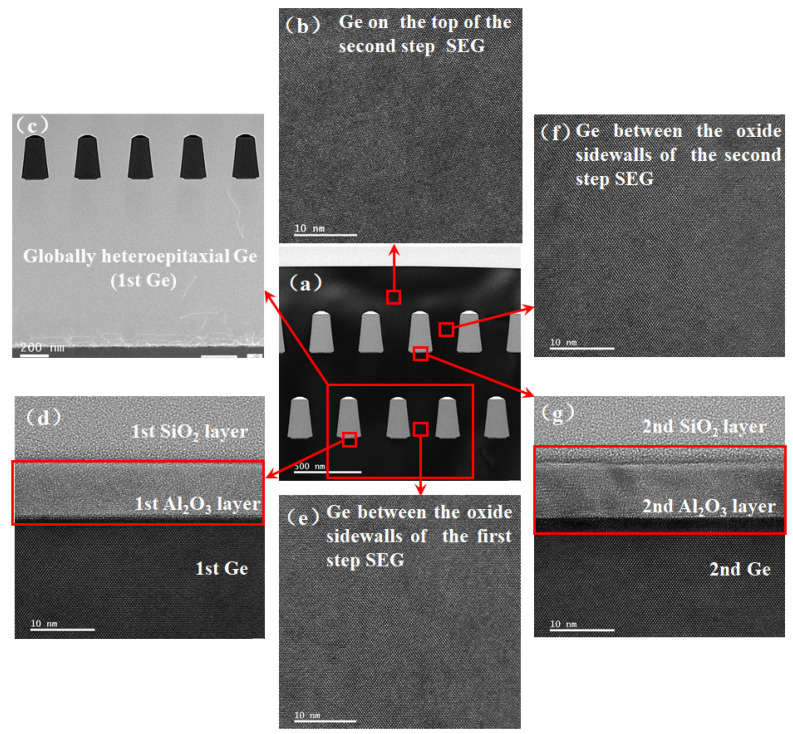
High-resolution TEM images taken at different positions of the film: (**a**) the whole film structure; (**b**) on the top region of the second SEG Ge layer; (**c**) the 1st Ge layer and the 2nd Ge layer; (**d**) the Al_2_O_3_ layer lies in the 2nd Ge layer; (**e**) near the bottom of the 2nd Ge layer; (**f**) in the middle of 3rd Ge layer; (**g**) the Al_2_O_3_ layer lies in the 3rd Ge layer.

The HRTEM was performed to further investigate the crystal quality of the SEG Ge epilayer. Figure 3a shows a cross-section HRTEM bright-field image of the whole film structure stack. Figure 3b–f are the enlarged parts of different positions which are marked in a red square, as shown in Figure 3a. Figure 3b,e,f show the different positions of the SEG Ge layer. It is clear that the Ge atomic planes are well-arranged without obvious distortion. Figure 3c shows the morphology of the first Ge layer and the second SEG Ge layer. A large number of TDs are observed at the Ge/Si interface. The TDs are reduced in the first Ge layer due to the optimized growth methods. Figure 3d,g illustrate the two Al_2_O_3_ adhesive layers. The Al_2_O_3_ layer is of great uniformity. No atomic diffusion occurs at the interfaces.

**Figure 4:**
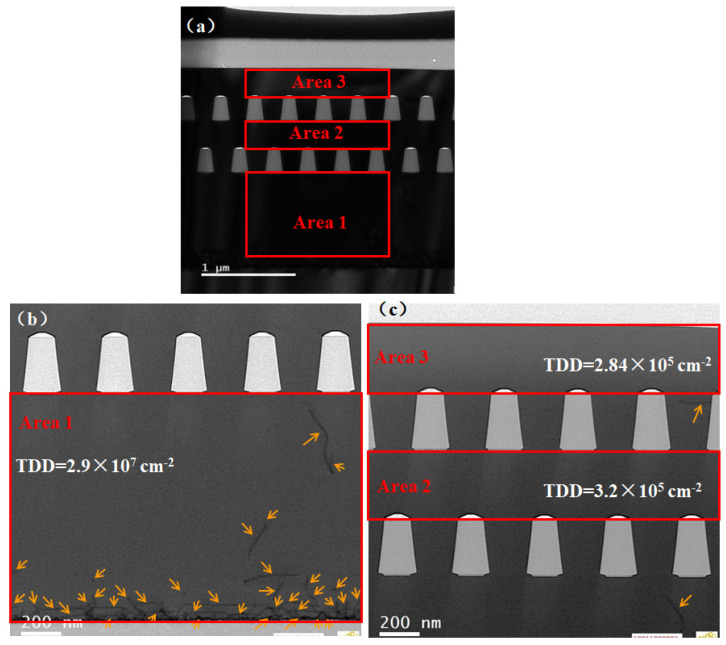
TEM images in typical areas of the film: (**a**) the whole film structure; (**b**) the 1st Ge layer and the 2nd Ge layer; (**c**) intermediate area between the first and second selective epitaxy.

To check the effect of the dual-step SEG Ge on the dislocations filtering, three typical areas, namely Area 1, Area 2 and Area 3, are selected to characterize TDs morphologies using HRTEM, as shown in Figure 4. Figure 4b depicts the dislocation distribution in Area 1 (the global heteroepitaxial Ge layer) and each dislocation is marked with an orange arrow. The density is high, close to the interface region due to the big lattice mismatch and thermal mismatch. The estimated TDDs of Area 1, Area 2 and Area 3 are 2.9 × 10^7^, 3.2 × 10^5^ and 2.84 × 10^5^ cm^−2^, respectively. Compared to the global heteroepitaxial Ge layer, the introduction of either one or two SEG Ge layers with oxide trenches help with reducing the TDDs by two orders of magnitude (from 10^7^ to 10^5^ cm^−2^). Dislocations propagating from the interface to the top of the global heteroepitaxial Ge are partially eliminated when they are encountered with oxide sidewalls. It is clear that the defects filtering effect of the first SEG step is highly efficient due to the relative dense distribution of defects in the globally heteroepitaxial Ge. However, the TDDs are basically at the same level in the second Ge layer. After the strain analysis, illustrated in the subsequent section, there is a partial strain relaxation in the second SEG epilayer which results in a rise of the TDD amount. This means that the new generated defects overshadow the filtering effect of the second-step SEG and the TDD reduction appears in minor scale.

**Figure 5:**
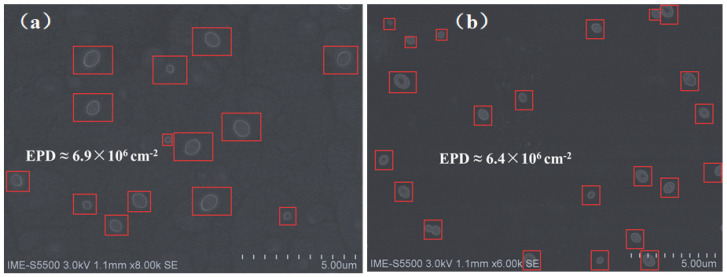
SEM images of the EPD samples: (**a**) single-step SEG Ge; (**b**) dual-step SEG Ge.

The etch pit density (EPD) method was also employed to evaluate the TDDs. The principle is that the corrosion rate near the dislocation area would be faster than that of the defects-free regions. After the etching process, deep pits/holes would appear at the dislocation area. To observe the defects in the Ge layer, the common etching solution composed of CH_3_COOH, HNO_3_, HF and I_2_ (iodine powder) is used [48]. The proportion is CH_3_COOH: HNO_3_: HF: I_2_ = 10 mL: 20 mL: 100 mL: 30 mg. A single-step SEG Ge sample and a dual-step SEG Ge sample were selected. Both samples were simultaneously immersed into the etching solution for 60 s and the etched surfaces were characterized by SEM, as illustrated in Figure 5. The etch pits are marked with a red square. The estimated TDDs of the single-step SEG Ge sample and the dual-step SEG Ge sample are 6.9 × 10^6^ and 6.4 × 10^6^ cm^−2^, respectively. These defect density results are larger compared to the result obtained using the TEM method. This variation is because the estimation of the EPD in the SEM is a global analysis method, whereas the TEM technique provides a local analysis method to determine the defect density. The former method uses chemical etching to create a contrast for the Eps over the sample surface; meanwhile, the latter method is a direct observation in smaller areas. However, these results confirm that the TDDs of the single-step SEG and dual-step SEG are quite similar.

**Figure 6:**
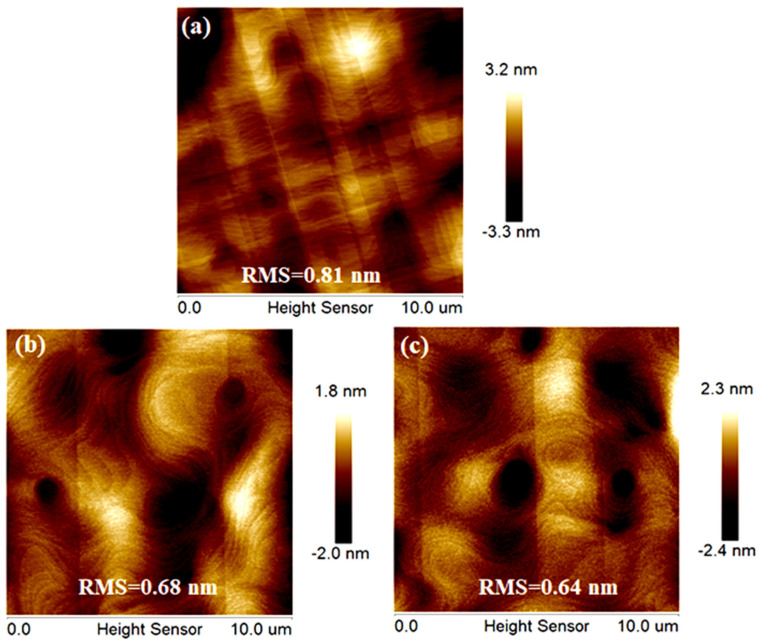
The 10 × 10 μm^2^ AFM images of different samples: (**a**) heteroepitaxial Ge; (**b**) single-step SEG Ge; (**c**) dual-step SEG Ge.

Figure 6 displays the 10 × 10 μm^2^ surface roughness by the AFM method. Figure 6a–c correspond to the samples of the global heteroepitaxial Ge, single-step SEG Ge and dual-step SEG Ge. The measured RMSs are 0.81, 0.68 and 0.64 nm, respectively. The surface roughness decreases gradually with the increase of SEG Ge layer. The dual- step SEG Ge sample has the minimum RMS value. However, the RMS of the dual-step SEG Ge sample is comparable to the single-step SEG Ge sample. Both of them have made great progress compared with the global heteroepitaxial Ge. The results indicate that the introduction of one SEG Ge layer has a remarkable efficacy to obtain a high-quality Ge surface. The introduction of one more SEG Ge layer would not lead to a significant improvement to the surface quality.

**Figure 7:**
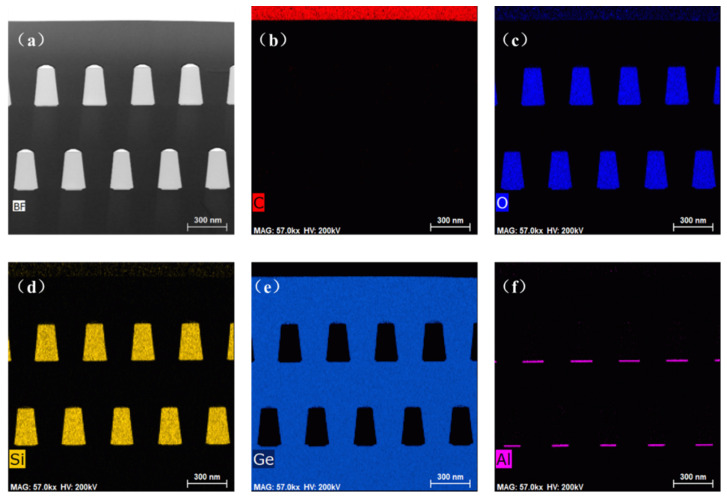
EDS characterization of the element distribution profiles: (**a**) bright-field image of the film structure, (**b**) C, (**c**) O, (**d**) Si, (**e**) Ge and (**f**) Al.

Figure 7 displays the elemental characterization by the EDS method. Figure 7a shows the bright-field image of the film structure. Figure 7b–f show the C, O, Si, Ge and Al element distribution profiles, respectively. It is clear that the boundary of each structure is consistent with the design, and no atomic diffusions are observed. Thus, it is an effective and reliable way to prepare low-TDD Ge material using the dual-step SEG Ge method.

### 3.2. Strain Characterization

**Figure 8:**
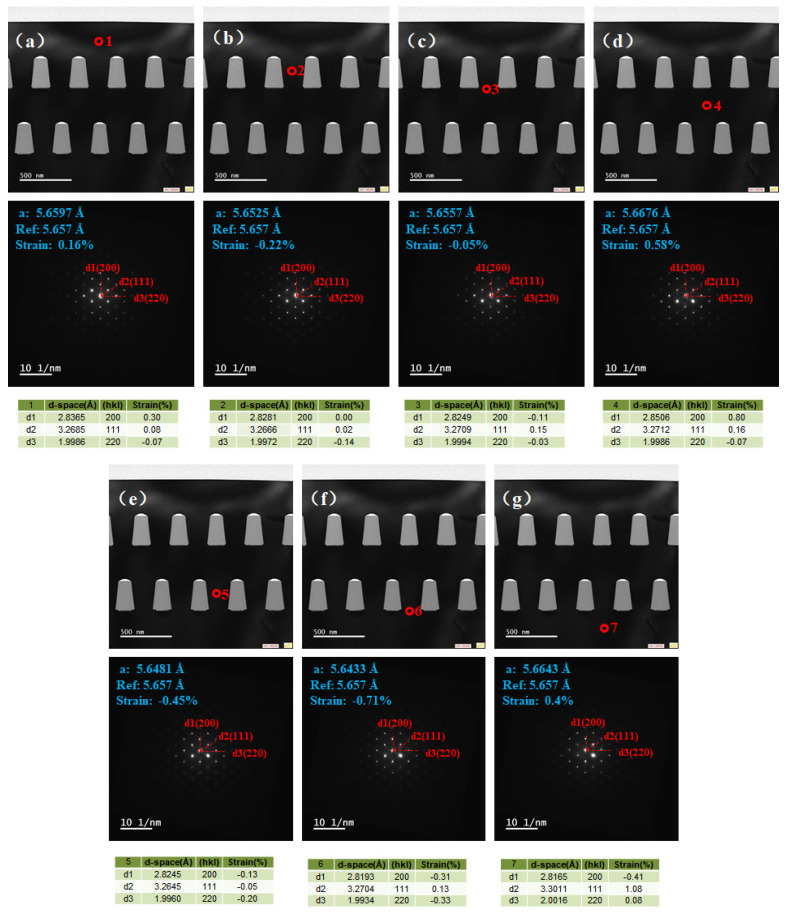
Strain distribution of Ge at different positions reflected by SAED: (**a**) Ge on the top of second-step selective epitaxial film; (**b**) Ge in second-step selective epitaxial trenches; (**c**) Ge at the bottom of second-step selective epitaxial film; (**d**) Ge on the top of first-step selective epitaxial film; (**e**) Ge in first-step selective epitaxial trenches; (**f**) Ge at the interface between the first-step selective epitaxial film and global heteroepitaxial film; (**g**) global heteroepitaxial Ge.

The selected area electron diffraction (SAED) patterns were taken at different positions along the [001] direction to analyze the strain states of the sample, as shown in Figure 8a–g. The patterns indicate good single-crystalline features. The following equation was used to calculate the lattice space *d*.

*Rd* = *Lλ* (1)

where *R* is the length of the camera, *λ* is the wavelength of the incident light and *L* is the spacing of the crystal planes in accordance with the diffraction bands. The *d*-values of the different positions in the sample were recorded in the tables. Then, the lattice constant *a* was deduced with the obtained lattice space values. The lattice constant *a* of different positions marked 1–7 are 5.6597 Å, 5.6525 Å, 5.6557 Å, 5.6676 Å, 5.6481 Å, 5.6433 Å and 5.6643 Å, respectively.

Taking into account that the fully relaxed Ge has a lattice constant of 5.657 Å (Ref value in Figure 8), the strain values at positions 1–7 were calculated to be +0.16, −0.22, −0.05, +0.58, −0.45, −0.71 and +0.4%, respectively. The +/− stands for the tensile/compressive strain. The compressive strain exists in the Ge layer between the SiO_2_ trenches (position 2 and 5) and near the bottom of the SiO_2_ trenches (position 3 and 6). Meanwhile, the tensile strain exists in the Ge layers above or below the SiO_2_ trenches, see positions 1, 4 and 7. The variation of the strain was modulated by the SiO_2_ trenches and the voids. Owing to the difference in the thermal expansion coefficient between the Ge (5.8 × 10^−6^ K^−1^) and the SiO_2_ (0.5 × 10^−6^ K^−1^), the compressive strain was introduced into the Ge material between the SiO_2_ trenches when the sample was cooled from the growth temperature to the room temperature. The compressive strain is favorable for the boost of the transport characteristics of the pMOS devices. Moreover, the SEG Ge layer was grown on a homogeneous Ge virtual substrate, which naturally eliminates the issues related to the mismatch in the parallel direction, such as different lattice constants and thermal performances. It should be mentioned that by introducing another SEG Ge layer, the compressive strain was improved from −0.53 to −0.71% near the same position compared to the single-step SEG Ge sample. It may be ascribed to the accumulative impacts of the thermal progress and the voids generated by the new SEG Ge layer.

**Figure 9:**
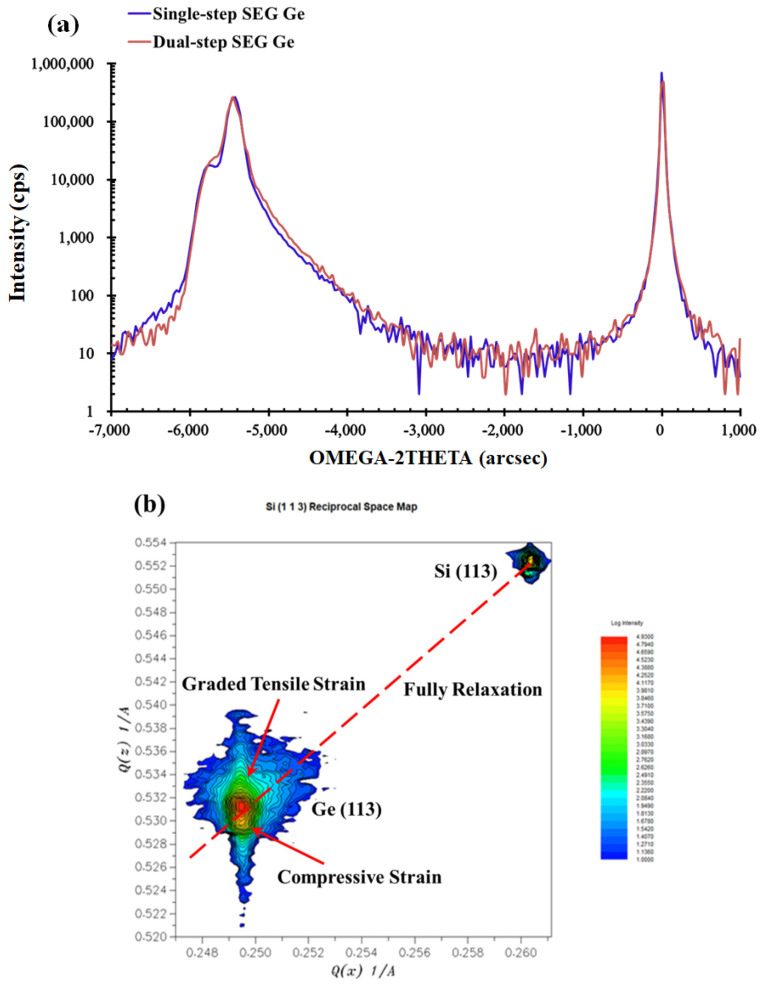
HRXRD spectrum: (**a**) comparison of the rocking curves around (004) between the dual-step SEG Ge sample and single-step SEG Ge sample; (**b**) HRRLMs of the dual-step SEG sample around (113); the red line is the relaxation line.

High-resolution X-ray diffraction (HRXRD) and high-resolution reciprocal lattice mappings (HRRLMs) were also performed to analyze the strain state. Figure 9a illustrates the comparison of the (004) rocking curves between the dual-step SEG Ge and the single-step SEG Ge samples. The Ge peak of both samples splits into two peaks. The left peak indicates a compressive strain, while the right one indicates a tensile strain. The full-width at half maxima (FWHM) which indicates the magnitude of defects is hard to measure because the two Ge peaks stay too close to each other. The HRRLMs around the (113) reflection were collected, and compressive strain was confirmed for the Ge peak located beneath the fully relaxation boundary, as shown in Figure 9b.

**Figure 10:**
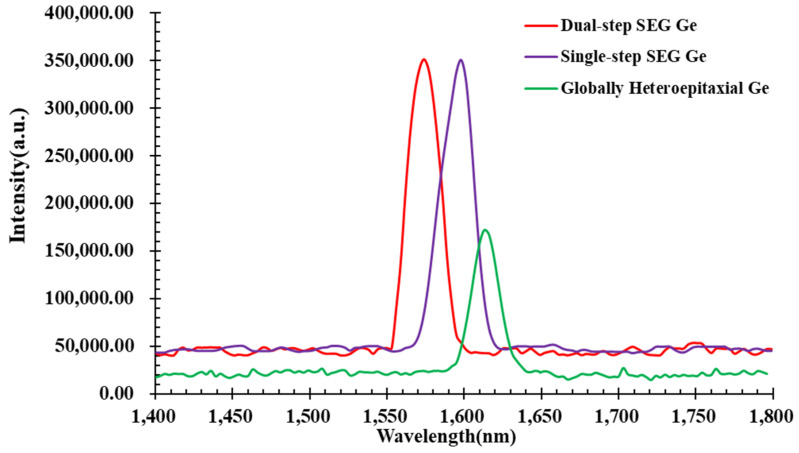
Room-temperature photoluminescence spectrum of samples: globally heteroepitaxial Ge, single-step SEG Ge and dual-step SEG Ge.

We also analyzed the crystal quality and the optical bandgap using the photoluminescence (PL) spectrum method. In general, the intensity of the PL spectrum is strongly related to the crystal quality. The spectrum intensity can be decreased by various factors, such as TDD, surface scattering, the recombination center caused by diffusion and surface non-radiative recombination centers. Figure 10 displays the room-temperature PL spectrums of the single-step SEG Ge sample and the dual-step SEG Ge sample using a 785 nm CW pumping laser. The wavelength peaks of the single- and dual-step SEG Ge samples are blue-shifted in contrast to the global Ge layer grown on the Si. This indicates a residual strain in both single and global epitaxy compared to the dual-step SEG Ge with fully strain relaxation.

## 4. Conclusions

This article has presented a novel method to significantly decrease the amount of TDD as well as to modulate the strain in Ge by using a dual-step selective epitaxy. The sample structures contained three epitaxy runs, starting with a globally grown Ge layer and two selectively grown Ge layers, where the oxide layer was deposited and patterned in two periods. The TDD was decreased consequently to 3.2 × 10^5^ cm^−2^ for the single-step SEG and to 2.84 × 10^5^ cm^−2^ for the dual-step SEG. The TDD has decreased by two orders of magnitude in the first SEG step and 11.3% in the second SEG owing to the defect-depleting effect. The ultimate RMS goes from 0.81 down to 0.64 nm as well. The minor decrease in the TDD in the second SEG of the Ge could be due to the strain relaxation which is a counterpart to the good effect of the defect-depleting effect. The strain was modulated from tensile strain to compressive and strain relaxed in the Ge cap layer. We believe that the dual-step SEG approach presented in this work provides a promising process for CMOS in the future, where both tensile and compressive strain are sought in the channel layer.

## Data Availability

The data is available on reasonable request from the corresponding author.
